# Acute right iliac fossa pain: not always appendicitis or a caecal tumour: two case reports

**DOI:** 10.1186/1757-1626-2-88

**Published:** 2009-01-27

**Authors:** Susim Kumar, Gerard J Fitzmaurice, Mark E O'Donnell, Robin Brown

**Affiliations:** 1 Department of General Surgery, Daisy Hill Hospital, Newry BT35 8DR, Northern Ireland, UK; 2 School of Health Sciences, University of Ulster, Jordanstown Campus, Shore Rd, Newtownabbey BT37 0QB, Northern Ireland, UK

## Abstract

**Background:**

A solitary diverticulum of the caecum is a rare benign condition which was first described by Potier in 1912 [1]. Clinical symptoms are usually a manifestation of complications arising from inflammation, perforation or haemorrhage. Despite radiological imaging, a pre-operative diagnosis is infrequent.

**Case presentation:**

We report two cases of right iliac fossa pain associated with a solitary caecal diverticulum. We discuss the clinical presentation, investigative modalities, and current therapeutic guidelines associated with this rare condition and highlight the difference from the more common conditions of appendicitis in the young and caecal neoplasms in the older patient.

**Conclusion:**

Complications of a solitary caecal diverticulum should be considered in the differential diagnosis of acute right lower quadrant pain. Mild caecal diverticulitis verified pre-operatively by radiological imaging or laparoscopically can be ameliorated by antibiotics alone. However, severe inflammation, perforation, haemorrhage or torsion necessitates a localised or radical resection. The presence of multiple diverticula, caecal phlegmon, or the inability to rule out an underlying caecal neoplasm warrants a right hemicolectomy.

## Background

A solitary diverticulum of the caecum is a rare benign condition which was first described by Potier in 1912 [1]. Although higher incidences have been reported in the Asian population, the condition still remains rare in the Western World [2]. Caecal diverticula are usually congenital in nature and arise as an out-pouching of the caecum involving all layers of the colonic wall [2]. They are usually asymptomatic unless complicated by inflammation, perforation or haemorrhage where presentation may mimic acute appendicitis with pyrexia, right lower abdominal pain and leucoytosis. Pre-operative diagnosis is invariably difficult even after radiological imaging. Therapeutic management varies from conservative treatment with antibiotics to surgical intervention ranging from diverticulectomy or wedge resections for local complications to right hemicolectomy in the presence of severe inflammation or if an underlying caecal neoplasm cannot be excluded.

## Case presentation

### Case 1

A 17-year old caucasian female presented with a 24-hour history of progressively increasing central abdominal pain which localised to the right iliac fossa. She had no other symptomatology and was otherwise well. On examination, the patient was mildly distressed and flushed. Her pulse was 90/minute, blood pressure was 120/60 mmHg, oxygen saturations were 99% on room air and her temperature was 36.4°C. Her abdomen was soft with maximal tenderness in the right iliac fossa and associated guarding. Rovsing's sign was positive. Haematological investigations demonstrated a haemoglobin level of 8.7 g/dl, white cell count of 8.1 × 10^9 ^/litre, and a C-reactive protein of 89.5 mg/L. Urinalysis was normal and a pregnancy test proved negative.

**Figure 1:**
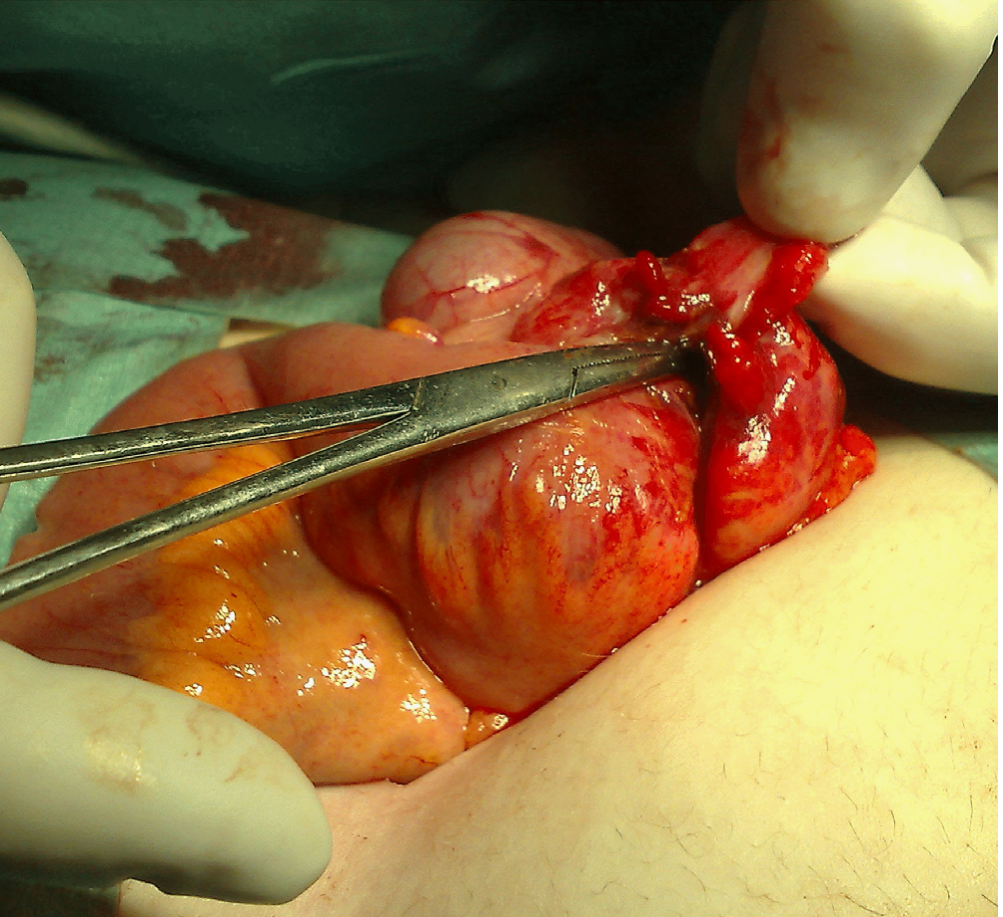
**Intraoperative view of the perforation through a solitary caecal diverticulum**.

She proceeded to appendicectomy via a low Lanz muscle-splitting incision. A faeculent smell was apparent on entering the peritoneal cavity. The appendix appeared injected with adjacent thickening of the ileocaecal region. A routine appendicectomy was performed. A small perforation was identified in the caecal wall just distal to the area of thickening at the ileocaecal area (Figure 1). The perforation was opened and explored as primary closure was deemed unsuitable. Although the mucosa appeared normal, the surrounding tissue was oedematous and friable and did not appear to be related to the appendicitis. A localised resection with preservation of the ileocaecal valve was performed with a two-layer closure using 3/0 polydioxanone (PDS) sutures. A saline lavage of the peritoneal cavity was completed and the wound was closed in layers. A subcutaneous Yeats drain was left in situ due to the initial faeculant spillage.

The patient was commenced on intravenous antibiotics three times per day (cefuroxime 1.5 g, metronidazole 500 mg and ampicillin 1 g). The patient made an uneventful recovery and was discharged on day-4 post-surgery. Histopathology confirmed the presence of a perforation within a solitary caecal diverticulum with evidence of extensive abscess formation extending into the adjacent fat with associated inflammatory infiltrates and fibrosis. There was no evidence of associated inflammatory bowel disease, dysplastic changes or malignancy.

### Case 2

A 47-year old caucasian male was admitted to hospital with a 3-week history of intermittent right lower abdominal pain with an exacerbation over the previous 4-days. He had associated anorexia, weight loss, lethargy and night sweats. The patient had no other relevant past medical history and was previously well prior to this episode. He was a smoker of 30 cigarettes per day. On examination, the patient was comfortable. He was haemodynamically stable (pulse 88/minute, blood pressure 130/85 mmHg) with a temperature was 37.8°C. His abdomen was soft with maximal tenderness in the right iliac fossa and right lumbar region where a tender mass was elicited. There was no peritonism. Rectal examination and rigid sigmoidoscopy to 15 cms were normal.

Haematological investigations demonstrated a haemoglobin level of 14.7 g/dl, white cell count of 11.1 × 10^9 ^/litre and an erythrocyte sedimentation rate of 69 mm/hour. Electrolyte and liver function tests, urinalysis and an ultrasound scan of the abdomen and pelvis were all normal. The patient was treated with carbalax suppositories and a barium enema revealed moderate diverticular disease of the ascending and descending colon with slight extrinsic compression on the medial aspect of the proximal ascending colon suggestive of a diverticular abscess. A contrast-enhanced CT scan of the abdomen and pelvis demonstrated no evidence of an abscess but an ill-defined 5 cm × 3 cm area projecting antero-laterally towards the right psoas muscle.

Due to deteriorating symptomatology, an exploratory laparotomy was performed which revealed a hard mass on the postero-medial aspect of the caecum. There was no other obvious intra-abdominal pathology. A right hemicolectomy was performed with a stapled (GIA 80 – Auto-suture) Barcelona type anastomosis. The suture line was oversewn and the mesenteric defect was closed.

The patient made an uneventful recovery and was discharged day-5 post-surgery. Histopathology revealed an inflammatory caecal pseudotumour with a diverticulum leading into the inflammatory mass. There was associated ascending colonic diverticulosis and no evidence of underlying inflammatory bowel disease or malignancy.

## Discussion

Solitary caecal diverticula (SCD) remain rare since their original description by Potier in 1912 [1]. There is a higher predominance in the Asian population compared to the Western nations where the incidence has been reported to be approximately 50 to 300 times less frequent than acute appendicitis equating to between 0.05 to 0.3 cases per 100,000 population [1-4]. Sardi *et al *(1987) reported a 3.6% prevalence of caecal diverticula from a review of 881 cases [1,5]. The average age at presentation was 43.6 (range 20–51) years with a male to female ratio of 3:2 [1,3]. Our first patient was atypical as she was only 17 years of age whereas our second was in his late forties.

A congenital aetiology for SCD in case 1 has been suggested due to a transient outpouching of the caecum in the 6^th ^week of gestation [1,3]. Caecal diverticula can be classified as solitary and multiple, and true (congenital) and false (acquired-with no muscle layer) [1,2]. SCD are usually situated approximately 2.5 cm from the ileo-caecal junction in 80% of cases with 50% of these located on the anterior caecal wall [3]. In case 2, the diverticulum entered into an inflammatory mass situated on the postero-medial aspect of the caecum.

A SCD is typically asymptomatic, only manifesting itself clinically when complicated by inflammation, haemorrhage, torsion or perforation [3,6]. Eighty-five percent of cases exhibit clinical features akin to appendicitis with right iliac fossa pain, low-grade pyrexia and a leucocytosis as observed in our first patient [2,5]. The absence of anorexia, infrequent nausea and vomiting and abdominal pain persisting for longer than 24-hours combined with a lack of systemic sepsis may help to differentiate SCD from appendicitis [2,7]. SCD rarely presents with a palpable mass as demonstrated in patient 2 where the actual mass effect was hypothesised to be related to repeated sub-clinical perforations followed by subsequent fibrin deposition. Based on histopathological analysis, it was suggested that this initiated an "onion-shell" type effect, where the repeated fibrin deposition formed a mass with numerous definable layers, which all tracked down to the SCD and presumed source of perforation. Invariably mis-diagnosed as appendicitis, other differentials to consider are urinary tract infection, ureteric colic, gastroenteritis, pelvic inflammatory disease, and Crohn's disease [2].

An abdominal x-ray may show a faecolith in about 50% of cases [2]. Although not indicated in the acute setting, a barium enema may demonstrate the diverticulum [2]. However, a SCD may also be missed due to obliteration of its lumen because of surrounding inflammation and oedema [3]. In a prospective study with 934 patients, Chou *et al *(2001) reported that ultrasound had a sensitivity of 91.3%, specificity of 99.8%, and an accuracy of 99.5% for the diagnosis of caecal diverticulitis in patients presenting with non-specific right lower abdominal pain [1,2]. The SCD appears as a hypo-echoic area on a portion of a thickened caecal wall [3]. However, ultrasound did not demonstrate this abnormality in case 2. Computerised tomography (CT) imaging is increasingly used especially in the acutely unwell patient. Classical CT features include a preserved enhancement pattern of the thickened caecal wall with an extra-luminal mass associated with haziness and linear stranding of the peri-caecal fat [3]. Laparoscopy has also been suggested especially in younger females with atypical symptomatology [1]. Despite advances in these investigative modalities, the majority of patients are diagnosed intra-operatively with a 65% to 85% accuracy for macroscopic diagnosis of SCD [1,2].

The treatment of caecal diverticulitis is controversial [2]. Conservative treatment with intravenous antibiotics can be considered if a definitive diagnosis is established pre-operatively [2]. If a SCD is clearly identified intraoperatively, a simple diverticulectomy or invagination of the diverticulum combined with appendicectomy have been advocated for uncomplicated diverticulitis [2,3]. Limited ileocaecal resections or right hemicolectomy should be considered in patients with marked inflammatory changes or if a complication such as perforation or torsion has occurred. However, 12.5% to 40% of patients undergoing conservative management or limited surgical resections are reported to require a more radical resection due to persistent or recurrent inflammation [2,6,8]. A right hemicolectomy is also mandatory if a diverticulum is macroscopically indistinguishable from a tumour especially if the SCD is retroperitoneally located on the posterior wall of the caecum [9]. This was advocated under similar circumstances in our second patient. This diagnostic conundrum can be surmounted by intraoperative caecoscopy, where an endoscope is guided through the appendicular stump to screen the mucosa for neoplasms [2]. Chiu *et al *(2002) emphasised the value of this endoscopic aid in excluding a caecal carcinoma, thereby allowing a more conservative resection of the colon in uncomplicated cases [2].

## Conclusion

Complications of SCD, though uncommon, should be considered in the differential diagnosis of acute right lower quadrant pain. Mild caecal diverticulitis verified pre-operatively by radiological imaging or laparoscopically can be ameliorated by antibiotics alone. However, severe inflammation, perforation, haemorrhage or torsion necessitates a localised or radical resection. The presence of multiple diverticula, caecal phlegmon, or the inability to rule out an underlying caecal neoplasm warrants a right hemicolectomy.

## Consent

Written informed patient consent was obtained from both patients for the publication of this case report and accompanying images. A copy of the written consent is available for review by the Editor-in-Chief of this journal.

## Competing interests

The authors declare that they have no competing interests.

## Authors' contributions

All authors have read and approved the final manuscript

SK: Involved in the literature review, manuscript preparation and manuscript editing

GF: Involved in literature review and manuscript preparation

MEOD: Involved in the conception of the report, literature review, manuscript preparation, manuscript editing, and manuscript submission

RB: Involved in the conception of the report, manuscript editing and manuscript review
